# Effect of Remote Ischemic Conditioning in Patients With Takotsubo Syndrome After Acute Stroke: Study Protocol for a Randomized Controlled Trial

**DOI:** 10.3389/fneur.2020.00286

**Published:** 2020-04-28

**Authors:** Tao Wang, Yueqiao Xu, Ning Wang, Meng Qi, Weitao Cheng, Xin Qu

**Affiliations:** Department of Neurosurgery, Xuanwu Hospital, Capital Medical University, Beijing, China

**Keywords:** stroke, remote ischemic conditioning, Takotsubo syndrome, randomized controlled trial, study protocol

## Abstract

**Introduction:** Takotsubo syndrome (TTS) is an acute heart failure syndrome which is preceded by a variety of emotional or physical triggers, with central nervous system conditions being an important trigger. Remote ischemic conditioning (RIC) is a promising interventional treatment based on the probability that both TTS and acute coronary syndrome may respond similarly to interventions. The heart protection effect of RIC has been repeatedly confirmed in animal models and observational clinical trials; however, it has never been studied in patients with TTS after acute stroke in randomized clinical trials with a higher level of evidence. The present study will be a proof-of-concept study to determine whether RIC can reduce cardiac injury and eventually improve the heart function and clinical outcomes of TTS patients after acute stroke.

**Methods and Analysis:** A single-center, outcome-assessor-blinded, randomized controlled trial (RCT) will be conducted to evaluate the effect of RIC in TTS patients after acute stroke. Major eligibility criteria include TTS patients diagnosed with acute stroke, which can be confirmed on computed tomography or magnetic resonance imaging; patients aged 18–75 years; patients admitted to a hospital within 48 h after the onset of acute stroke; and patients diagnosed with Takotsubo cardiomyopathy with an InterTAK diagnostic score ≥50. A total of 60 eligible patients will be randomly allocated into either the RIC or the control group. The primary endpoint is a composite of death from any cause and major adverse cardiac and cerebrovascular events during the in-hospital period and at the 1- and 6-month follow-up.

**Ethics and dissemination:** This study has been approved by the Medical Ethics Committee of Xuanwu Hospital, Capital Medical University ([2017] 072). The study findings will be presented at international conferences and published in a peer-reviewed journal.

**Trial registration:** This study has been prospectively registered in the Chinese Clinical Trial Registry on September 10, 2018 (ChiCTR1800018290).

## Introduction

### Description of the Disease

Takotsubo syndrome (TTS) represents an acute heart failure condition which is often preceded by sudden extreme stresses including emotional or physical triggers (1). A variety of physical stressors have been reported, including acute critical illness, acute respiratory failure, pancreatitis, traumatic injury, and iatrogenic factors such as surgery, exercise tests, diarrhea, or dobutamine stress echocardiography (1, 2). Central nervous system conditions such as stroke, traumatic brain injury, intracerebral hemorrhage, or seizures also represent an important trigger (1). Patients with TTS usually present with acute chest pain, breathlessness, palpitations, sweating, nausea, and congestive heart failure (1, 2) its clinical symptoms are similar to acute coronary syndrome, except demonstrable coronary artery stenosis or spasm (3). TTS is characterized by transient left ventricular dysfunction, which extends to more than one epicardial coronary artery territory, and a disproportionate increase in troponin compared with the diffuse electrocardiographic and cardiac imaging changes (4). Currently, there are no widely accepted diagnostic criteria for TTS. The most widely used criterium was the modified proposed criteria by the Mayo Clinic for the diagnosis of TTS (5). The InterTAK International Registry Group has developed a simple scoring system, the InterTAK diagnostic score (6), that takes into account five historically used clinical variables and two ECG variables. The InterTAK diagnostic score is slightly different from the modified Mayo Clinic criteria (7). Although considered as a fully reversible disease in most cases, TTS has been described in about 52% of cases to be associated with various complications, such as acute congestive heart failure, acute pulmonary edema, shock, and ventricular fibrillation, and it is in acute phase in most cases (8–11).

Several hypotheses have been proposed for the pathogenesis of TTS, including aborted myocardial infarction (MI) with spontaneous recanalization, myocarditis, coronary artery vasospasm, blood-borne catecholamine myocardial toxicity, and autonomic nervous system dysfunction with sympathetic hyperexcitation (12, 13), of which, the involvement of the adrenergic cascade triggered by physical or emotional stress is considered to be the most convincing (14, 15). Additionally, catecholamines can exert a direct catecholamine toxic effect on myocytes, involving the oxidative stress and inflammatory cascades (16), which can cause microvascular dysfunction, thus leading to reduced myocardial perfusion (17).

### Description of the Intervention

Previous studies revealed that both neuronal and humoral components are involved in the signal transfer in remote ischemic conditioning (RIC) treatment; they act in concert and interact on three different levels from the stimulation site to the target organ. Firstly, peripheral sensory nerves are directly activated by the ischemic/reperfusion stimuli and/or indirectly activated by the humoral factors (18). Secondly, the peripheral sensory afferent nerves projecting to the autonomic centers of the central nervous system eventually activate efferent vagal nerves (18), which subsequently release acetylcholine to directly activate receptors in the target organs and/or activate receptors in other organs that can release humoral factors to indirectly act on the target organs (19). Finally, the intrinsic nervous system and the response of the specific parenchymal cells of the target organ are activated to contribute to the final effect (19, 20). Though transient receptor potential vanilloid 1 (TRPV1), calcitonin gene-related peptide (CGRP), substance P, and other mediators were found to be involved in RIC, the exact mechanisms of these pathways are still far from clear (18, 21–23). In this present study, a preliminary exploration on the changes in cardiac biomarkers will be made after RIC treatment; however, more basic research is still required to clarify the specific mechanism.

### Rationale for the Present Study

Though the role of myocardial stunning in the mechanism of TTS is still unclear, some therapeutic algorithms have been developed for the treatment of TTS patients (1) based on the probability that both TTS and acute coronary syndrome may respond similarly to pharmacological or other interventions (24), among which, RIC is a promising intervention treatment. Ischemic conditioning was first recognized in 1986, when it was discovered that brief interruption of blood supply to a vascular territory limits the extent of ischemia, reperfusion injury, and the eventual size of the MI (25). Soon after, it was found that the brief and complete interruptions of blood supply to a particular vascular territory provide benefits to other vascular territories to a certain extent (26–29). Since then, the heart protection effect of RIC has been repeatedly confirmed in various animal models (30–36). Furthermore, several clinical studies have also shown the beneficial effects of RIC on heart injuries, such as ST-segment elevation myocardial infarction (37, 38), and heart failure after primary percutaneous coronary intervention (39). However, the heart protection effects of RIC have not been observed in all patients with heart injuries. Kahlert et al. reported that RIC provided no protection effect on the heart before transfemoral transcatheter aortic valve implantation in the interim analysis of a randomized controlled trial (RCT) (40). In particular, RIC has never been studied in patients with TTS patients after acute stroke. Therefore, whether RIC can benefit TTS patients after acute stroke still needs to be clarified.

### Objectives

The present study will be a proof-of-concept study to determine whether RIC can exert a heart protection effect and thereby reduce cardiac injury and eventually improve the heart function and clinical outcomes of TTS patients after acute stroke.

## Methods and Analysis

### Overall Design

**Figure 1:**
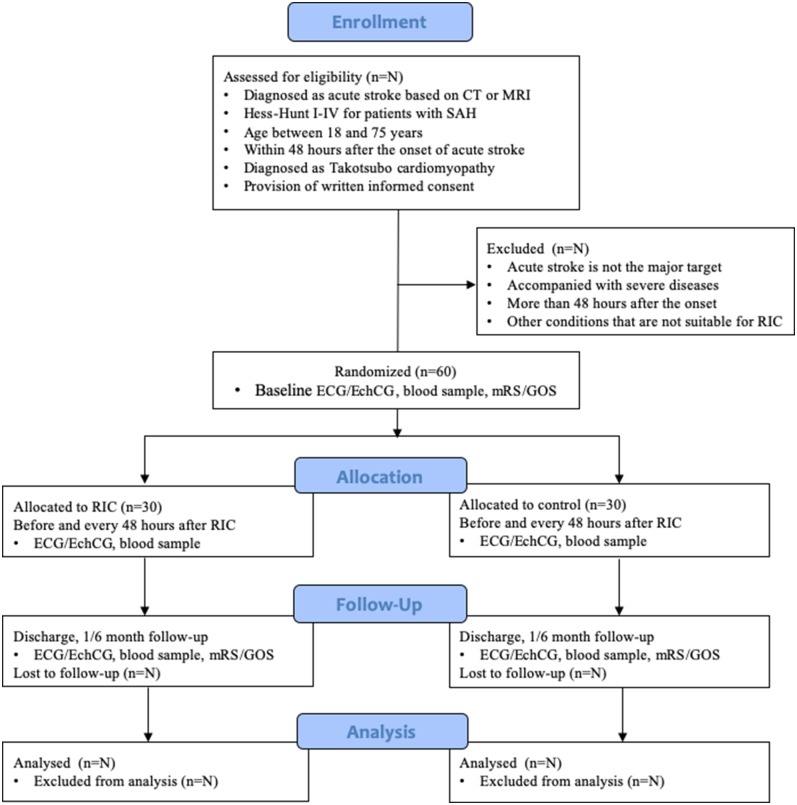
Study flow diagram according to CONSORT 2010. *SAH*, subarachnoid hemorrhage; *RIC*, remote ischemic conditioning; *ECG*, electrocardiograph; *EchCG*, echocardiograph; *mRS*, modified Rankin Scale; *GOS*, Glasgow Outcome Score.

A single-center, outcome-assessor-blinded, randomized controlled trial will be conducted in the Neurosurgical Intensive Care Unit (NICU) of Xuanwu Hospital, a tertiary neurosurgical referral centre in Beijing, China, and aims to evaluate the effect of RIC on TTS in patients with acute stroke. This protocol has been developed according to the Standard Protocol Items: Recommendations for Interventional Trials (SPIRIT) (41). The conduction and reporting of this RCT will follow the Consolidated Standards of Reporting Trials (CONSORT) statement and its extension to non-pharmacological interventions (42). The study process is summarized in Figure 1.

### Eligibility Criteria

Adult patients with acute stroke referred to the NICU will be screened for trial eligibility. Routine ECG, echocardiography, and serum examination of cardiac injury biomarkers will be performed. Based on the InterTAK diagnostic score (6), patients diagnosed with TTS (InterTAK diagnostic score ≥50) will be recruited into this study. At least two physicians will review the eligibility criteria, and a third senior physician will resolve any disagreement. The inclusion and exclusion criteria are summarized as follows:

#### Inclusion Criteria

1. Patients diagnosed with acute stroke (ischemic or haemorrhagic), which can be confirmed on computed tomography (CT) or MRI;
2. Patients diagnosed with subarachnoid hemorrhage with Hess–Hunt grades I–IV;
3. Patients aged 18–75 years;
4. Patients admitted to hospital within 48 h of acute stroke onset;
5. Patients diagnosed with Takotsubo syndrome with an InterTAK diagnostic score ≥50 (6). The ventricular wall motion will be evaluated with echocardiography. If acute coronary syndrome (ACS) is suspected based on ECG, echocardiography, or biomarker findings, coronary computed tomography angiography (CTA) will be conducted to rule out ACS. Coronary angiography and cardiac magnetic resonance imaging will be utilized to differentiate ACS only when necessary.
6. Patients with a signed written informed consent form.

#### Exclusion Criteria

1. Acute stroke is not the major cause for admission or the patient is receiving palliative care.
2. The patients suffer from concurrent diseases, such as severe cardiovascular disease, are in an extremely critical condition, have cerebral hernia, and/or it is highly expected that the patients will fail to complete the study.
3. The patients are admitted to the hospital more than 48 h after acute stroke onset or have any subsequent stroke after the diagnosis of TTS.
4. The patients have concurrent limb vascular occlusive disease and/or other diseases or conditions which cause intolerance to the intervention treatment.
5. Both upper limbs of the patients are affected by the stroke, indicated by any symptoms, abnormal results of physical examination, or neurophysiologic tests in any upper limbs.
6. The patients have other conditions that are not suitable for RIC treatment, such as skin damage, severe thrombocytopenia, and abnormal blood clotting.

### Randomization and Blinding

A total of 60 eligible patients with acute stroke, in accordance with the inclusion and exclusion criteria, will be included in this study. Random numbers will be generated on a computer by an unblinded physician who will not be involved in the data analysis and then placed in sealed envelopes. The patients will be randomly allocated into either the RIC or the control group according to a ratio of 1:1. The physician responsible for performing RIC will not be blinded and will play no further role in the clinical trial; the staff responsible for the data collection and analysis will be blinded. Hence, this is an outcome-assessor-blinded study.

### Intervention and Follow-Up

All eligible patients in the RIC and control groups will receive routine monitoring and medical treatment. Moreover, the patients in the RIC group will receive RIC treatment through an electric auto-control device that has been delineated by Ji et al. (patent number ZL200820123637.X, China): (43) a blood pressure cuff will be placed around the upper arm of the patient and inflated to a pressure of 200 mmHg (44) for 5 min and then deflated for reperfusion for 5 min. The inflation and deflation will be performed for four cycles each time, three times a day for a total of five consecutive days. If an upper limb is affected by the stroke, the other normal upper limb will be selected for RIC treatment. Blood samples will be collected before RIC treatment, every 48 h after RIC treatment until discharge from the NICU. The patients in the control group will not receive RIC treatment; however, blood samples will be collected at the same time points as those in the RIC group. Blood samples will be used for the detection of the following biomarkers: cardiac troponin I (cTnI), N-terminal pro B-type natriuretic peptide (NT-pro BNP), high-sensitivity C-reactive protein (HS-CRP), interleukin-6 (IL-6), interleukin-10 (IL-10), interleukin-17 (IL-17), plasminogen activator inhibitor-1, plasma tissue plasminogen activator, micro-RNA-126, micro-RNA-155, serum nitric oxide (NO), blood uric acid, white blood cell count, and platelet aggregation rate. The electrocardiograph and echocardiography will be performed simultaneously every 48 h after RIC treatment until discharge from the NICU to evaluate the cardiac indicators such as ECG ST segment, percent of arrhythmia, ejection fraction, and left ventricular end-diastolic diameter (LVEDD). All eligible patients will be followed up by core team members based on clinical visits, medical records, or telephone interviews at 1 and 6 months after discharge and undergo the aforementioned examinations and serological tests (Table 1).

**Table 1:** Data collection timeline.

	**Cardiac examination (electrocardiograph, echocardiography)**	**Blood samples**	**Patients' characteristics (mRS or GOS)**	**Clinical outcomes**
At admission	✓	✓	✓	
Before RIC	✓	✓		
Every 48 h after RIC	✓	✓		
At discharge from NICU	✓	✓	✓	✓
At 1 month after discharge	✓	✓	✓	✓
At 6 months after discharge	✓	✓	✓	✓

*RIC, remote ischemic conditioning; NICU, neurosurgical intensive care unit; mRS, modified Rankin Scale; GOS, Glasgow Outcome Score*.

### Quality Control and Safety Assessment

We will develop a manual describing in detail all aspects of the recruitment, eligibility criteria, randomization, interventions, data collection, and case record form (CRF) completion. All staff involved in this trial will be trained beforehand. This manual will be utilized to monitor adherence to procedural steps. Each cuff inflation/deflation time will be recorded to monitor adherence to the intended intervention. The study process will be supervised by an independent data monitoring committee. RIC is a non-invasive means to treat TTS patients by performing repeated inflation and deflation of a blood pressure cuff around the upper arm. However, it should be taken into account that there will be some possible adverse events, such as transient discomfort and/or ischemic damage of the upper arm during compression of the cuff, the formation of ecchymosis on the upper arm after RIC treatment, and new or aggravating neurological deficits during or after RIC treatment. Any serious adverse events possibly related to RIC treatment will be reported to the ethical committee.

### Patient and Public Involvement

The protocol of this study was discussed and developed by a multidisciplinary team of experts including epidemiologists, clinicians, and statisticians; however, no patients or public people were involved in the design phase.

### Primary Endpoints

The primary endpoint is a composite of death from any cause and major adverse cardiac and cerebrovascular events (a composite of a recurrence of Takotsubo syndrome, myocardial infarction, stroke or transient ischemic attack, or death from any cause) evaluated during the in-hospital period and at the 1- and 6-month follow-up.

### Secondary Endpoints

The secondary endpoints are death from any cause, a composite of major adverse cardiac and cerebrovascular events, serum cardiac biomarker cTnI, NT-pro BNP, modified Rankin Scale (mRS) score, Glasgow Outcome Score (GOS), cardiac function determined by echocardiography, ECG changes (QT interval and possible ST elevation), and blood uric acid level measured prior to RIC treatment, at discharge from the NICU, and at the 1- and 6-month follow-up. The average hospitalization duration will be recorded as a secondary endpoint to evaluate the efficiency of RIC.

### Sample Size

No previous studies have focused on utilizing RIC to prevent TTS in patients after acute stroke. Most existing evidence utilized troponin I assay for the detection of cardiac injury. Power calculations for this proof-of-concept study will be based on serial troponin measurements, taken as continuous outcome variables with a predicted difference. Previous studies on RIC indicated a cardioprotective effect of RIC in reducing troponin levels by 18–62% (45–48). Based on the results of a pilot study in our center (the troponin level decreased by 40%, *n* = 10), we hypothesized a troponin level reduction of 37% with 80% power at the 5% significance level, and we calculated a sample size of 60 patients (*n* = 30 in each arm of the study).

### Data Management and Statistical Analysis

An electronic database will be established to collect the data. Unique numeric identifiers will be assigned to each patient, which will be included in the study database; any other information related to the patient's identity will be excluded. Patients' identification will be solely used for follow-up purposes. Data safety will be supervised by an independent monitoring committee. Descriptive and summary statistics will be calculated for each treatment group for baseline characteristics. Continuous data will be expressed as means (standard deviations) or medians (interquartile ranges) for non-normal continuous data and ordinal data. Primary and secondary endpoints will be analyzed by the intent-to-treat principle. In univariate analyses, patient- and clinical-related factors will be compared *via* the chi-square, analysis of variance, Student's *t*-test, or Mann–Whitney *U* test, when appropriate, between two groups. The variables in the initial model included age, gender, diabetes status, hypertension, smoking status, serum cardiac biomarkers, blood uric acid, ejection fraction, LVEDD, mRS, and GOS at baseline. The relative prognostic significance of the variables in predicting the primary endpoint, mRS and GOS will be assessed by multivariate Cox regression models. Variables associated with a *p* < 0.10 on univariable analysis were included in the multivariable analysis. Kaplan–Meier plots and log-rank tests will be utilized to compare the mRS and GOS between two groups. A *p* < 0.05 will indicate statistical significance. SPSS 13.0 software (SPSS Inc., Chicago, IL) will be used for all statistical analyses.

## Discussion

We described the design and methods of a single-center, two-arm, parallel-group, randomized controlled trial investigating the efficacy of RIC in patients with TTS after acute stroke. This is the first RCT to address important questions about the heart protection effect of RIC in this population with such rigorous methodological design. It is expected that the study findings might hopefully become part of local protocols or international guidelines, allowing patients and care providers to make informed decisions and provide comprehensive information for future study designs.

A limitation of this study is that it involves only one center and a minority of the subjects with TTS. This might influence the generalizability of the research results. Another limitation of this study is the non-blinding of the physician responsible for performing RIC. To reduce potential influence of this limitation on the outcomes, the physicians have no further role in the clinical trial and the staff responsible for data collection and analysis will be blinded.

## Ethics and Dissemination

This study has been approved by the Medical Ethics Committee of Xuanwu Hospital, Capital Medical University ([2017] 072), and the study findings will be presented at international conferences and published on a peer-reviewed journal. Safety considerations will be addressed as previously described.

## Ethics Statement

This study protocol has confirmed with the declaration of Helsinki and has been reviewed and approved by the Medical Ethics Committee of Xuanwu Hospital, Capital Medical University ([2017] 072). All patients and/or their proxies will be asked to sign a written informed consent form.

## Author Contributions

YX was the principal investigator, has initiated the study, and applied for funding. TW was the study coordinator and has been involved in the study design and drafted this manuscript. NW, MQ, WC, and XQ have been involved in the conception and study design. MQ has made important statistical contributions. All authors provided feedback on drafts of this paper and read and approved the final manuscript.

## Dissemination Policy

The researchers of this study have the intention to disseminate the results of the trial as widely as possible. Firstly, the trial has been registered on international clinical trial register database (registration number: ChiCTR1800018290, http://www.chictr.org.cn/showproj.aspx?proj=30637). Secondly, this study protocol will be published on a peer-reviewed journal. Finally, the findings will be presented at international meetings and disseminated to patients through peer-reviewed publications and newsletters. The identity of the patients will not be disclosed in any of these publication forms. The researchers of this study will attempt to publish the study results as soon as possible.

## Conflict of Interest

The authors declare that the research was conducted in the absence of any commercial or financial relationships that could be construed as a potential conflict of interest.

## Abbreviations

TTS: Takotsubo syndrome
SAH: subarachnoid hemorrhage
ECG: electrocardiograph
MI: myocardial infarction
RIC: remote ischemic conditioning
RCT: randomized controlled trial
HS-CRP: high-sensitivity C-reactive protein
IL-6: interleukin-6
IL-10: interleukin-10
IL-17: interleukin-17
LVEDD: left ventricular end-diastolic diameter
CRF: case record form
mRS: modified Rankin Scale
GOS: Glasgow Outcome Score
ChiCTR: Chinese Clinical Trial Registry
TRPV1: transient receptor potential vanilloid 1
CGRP: calcitonin gene-related peptide.
